# rAAV immunogenicity, toxicity, and durability in 255 clinical trials: A meta-analysis

**DOI:** 10.3389/fimmu.2022.1001263

**Published:** 2022-10-27

**Authors:** Weiran Shen, Shengjiang Liu, Li Ou

**Affiliations:** ^1^ Obio Technologies, Shanghai, China; ^2^ Avirmax Inc, Hayward, CA, United States; ^3^ Genemagic Biosciences, Wallingford, PA, United States; ^4^ Department of Pediatrics, University of Minnesota, Minneapolis, MN, United States

**Keywords:** rAAV, clinical trials, immunogenicity, toxicity, neutralizing antibodies, immunosuppressants, capsids, gene therapy

## Abstract

Recombinant Adeno-associated virus (rAAV) is one of the main delivery vectors for gene therapy. To assess immunogenicity, toxicity, and features of AAV gene therapy in clinical settings, a meta-analysis of 255 clinical trials was performed. A total of 7,289 patients are planned to be dosed. AAV2 was the most dominantly used serotype (29.8%, n=72), and 8.3% (n=20) of trials used engineered capsids. 38.7% (n=91) of trials employed neutralizing antibody assays for patient enrollment, while 15.3% (n=36) used ELISA-based total antibody assays. However, there was high variability in the eligibility criteria with cut-off tiers ranging from 1:1 to 1:1,600. To address potential immunogenicity, 46.3% (n=118) of trials applied immunosuppressants (prophylactic or reactive), while 32.7% (n=18) of CNS and 37.5% (n=24) of ocular-directed trials employed immunosuppressants, possibly due to the immune-privileged status of CNS and retina. There were a total of 11 patient deaths across 8 trials, and 18 out of 30 clinical holds were due to toxicity findings in clinical studies. 30.6% (n=78) of trials had treatment-emergent serious adverse events (TESAEs), with hepatotoxicity and thrombotic microangiopathy (systemic delivery) and neurotoxicity (CNS delivery) being the most prominent. Additionally, the durability of gene therapy may be impacted by two distinct decline mechanisms: 1) rapid decline presumably due to immune responses; or 2) gradual decline due to vector dilution. The durability varied significantly depending on disease indication, dose, serotypes, and patient individuals. Most CNS (90.0%) and muscle trials (73.3%) achieved durable transgene expression, while only 43.6% of ocular trials had sustained clinical outcomes. The rAAV production system can affect rAAV quality and thus immunogenicity and toxicity. Out of 186 trials that have disclosed production system information, 63.0% (n=126) of trials used the transient transfection of the HEK293/HEK293T system, while 18.0% (n=36) applied the baculovirus/Sf9 (rBac/Sf9) system. There were no significant differences in TESAEs and durability between AAV generated by rBac/Sf9 and HEK293/HEK293T systems. In summary, rAAV immunogenicity and toxicity poses significant challenges for clinical development of rAAV gene therapies, and it warrants collaborative efforts to standardize monitoring/measurement methods, design novel strategies to overcome immune responses, and openly share relevant information.

## Introduction

Since the first clinical study using recombinant AAV (rAAV) in cystic fibrosis in the 1990s (1), great progress has been made in understanding its virology, production, safety, efficacy, and translatability (2). In the past 10 years, rAAV has been widely applied in treating rare diseases affecting liver, brain, muscle, heart, eye, and other tissues (3). Long-lasting transgene expression has been achieved in multiple trials, and a total of six therapies have received marketing approval from US Food and Drug Administration (Luxturna, Zolgensma) and European Medicines Agency (Glybera, Luxturna, Zolgenmsa, Upstaza, Roctavian) (4). However, the loss of efficacy is not uncommon in clinical trials, which may result from various reasons, including immune response against AAV (4–7). Moreover, immune responses against rAAV vectors include pre-existing antibodies, complement system activation, and T cell immune responses (8–12). Understanding the complex mechanisms of immune activation is difficult, and the data interpretation is further complicated by individual and species differences, and non-standardized outcome measurements in these studies. Serum prevalence studies suggested a significant portion of the human population had high levels of rAAV neutralizing antibodies (13). Pre-existing antibodies can significantly reduce the transduction efficiency (14). Antibodies bind to vector capsid and stimulate lymphocytes to secrete cytokines, activating adaptive immune responses (9). Thus, during patient enrollment in clinical studies (especially for systemic administration), pre-existing antibody levels need to be considered. After vector dosing, transduced antigen presenting cells (APCs) present capsid peptides by MHC-I or MHC-II system, and activate plasmacytoid and conventional dendritic cells (10, 11, 15). Also, rAAV genome is hypomethylated in CpG sequences (16), which activates toll-like receptor 9 (TLR9) (17). TLR9 -dependent and -independent signaling triggers MyD88 phosphorylation, which is a critical step in activation of cytotoxic T cells (CTLs) reactive to viral capsids (17–19). MyD88 also plays an important role in B cell activation and regulates the Th1-dependent antibody production against rAAV (20). A Hemophilia B trial suggests that CpG introduced by codon optimization might be a trigger for silenced transgene expression (21). A statistical study tried to correlate CpG content in genome sequences of clinical used products with efficacy. It also showed that lower CpG content may reduce the overall immune responses and be helpful to maintain the efficacy (22). Further suggestions are made on methods to calculate and mitigate CpG risk (23, 24). Interestingly, it is still unclear how much unmethylated CpG is a safe range. It is meaningful to develop a translatable model and test whether modification of CpG in gene of interest (GOI) is enough, or reducing CpG number in ITR and regulatory elements are also essential. Recent studies also reported that AAV may activate the complement system, causing platelet reduction, acute thrombotic microangiopathy (TMA) (25, 26). To reduce the potential immune responses and toxicity, immunosuppressants are selectively applied across different clinical trials (27). In this study, we performed a meta-analysis of 255 clinical trials using AAV delivery over the past 25 years. The immunosuppressant usage, antibody screening, adverse events, manufacturing systems, and durability of efficacy was evaluated. Results from this study will highlight the trend and new directions in this field, provide a basis for future clinical study design, and uncover crucial details for addressing immune responses against AAV.

## Methods

This meta-analysis was performed under the guidance of the 2020 PRISMA (28). Clinical trials using AAV delivery were extracted from the U.S National Library of Medicine database (ClinicalTrials.gov), and the cut-off date was June 1, 2022. Observational studies and long-term follow-up studies were not included because AAV administration was not involved. Additionally, the search was broadened to (1) company press releases (2), company official documents (SEC and IPO filings), (3) presentations in research conferences, (4) publications in journal articles, and (5) company websites. Since a lot of information was not available in the database, reports on previous preclinical studies using the same test article were also reviewed. Disease category, disease indication, gene delivered, trial start date, phase, identifier, serotype, dose, sponsor, route of administration (ROA), immunosuppression regimen, antibody screening, manufacturing system, adverse events, efficacy endpoints, and trial status were collected. Missing information was marked as “Undisclosed” or “N/A”. Notably, peer-reviewed resources and information from non-reviewed resources (e.g., websites, databases) were equally used in this study. All diseases were categorized as central nervous system (CNS), liver, lysosomal storage disorders (LSD), ocular, muscle, and other (including oncology, autoimmune, virology, cardiac, metabolic, pulmonary). All raw data is available in **Supplementary Table 1**.

## Results

### Description of 255 clinical trials

**Figure 1:**
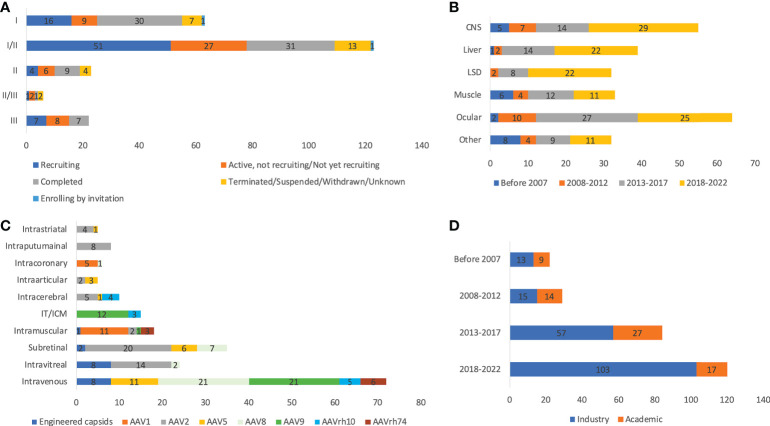
Basic information of 255 AAV clinical trials by status and phases **(A)**, disease indications **(B)**, ROAs and serotypes **(C)**, and sponsors **(D)**. ROA and serotypes associated with <10 trials are not included in the figure. IT and ICM were combined due to the high similarity. Unknown status suggests that study has passed its completion date and status has not been verified in more than two years.

A previous study reviewed a total of 149 AAV clinical trials from clinical trial databases using the cut-of date as Jan 1, 2020 (3). In that study, phases, serotypes, ROA, promoters used, and sponsors were analyzed. In this study, the cut-off date was expanded to June 1, 2022, and the sources included clinical trial databases, publications, and company press releases, resulting in analysis of 255 trials. The process of data collection was summarized using a PRISMA flowchart (**Supplementary Figure 1**). A total of 65 trials are in Phase I, 123 in Phase I/II, 25 in Phase II, 6 in Phase II/III, 22 in Phase III, and one in Phase IV (**Figure 1A**). There is a single trial (GNT0004 sponsored by Genethon) combining Phase I, II, and III studies together. A total of 245 trials report study status, with 79 ‘completed’, 81 ‘recruiting’, 50 ‘active but not recruiting’. There are also 18 trials ‘terminated’, 6 ‘unknown’, and 2 ‘withdrawn’. In terms of trial number, ocular indications constitute the highest portion (25.1%), followed by CNS (21.6%) and liver (15.3%) (**Figure 1B**). Across 255 trials, more than 7,289 patients are planned to be dosed. A total of 3,192 patients have been enrolled or dosed, while the largest study (NCT04704921) is still actively enrolling patients, targeting a total of 465 people. A total of 94 trials are designed to enroll at least 20 patients. There are 56 trials (22.0%) sponsored by universities, hospitals, or other academic institutes, while the rest (78.0%) are sponsored by industry. The organizations that sponsored the most trials are Spark Therapeutics, Novartis, Nationwide Children’s Hospital, REGENX Bio (n=10, 9, 8, and 8, respectively). In terms of ROA, 92 trials apply systematic injection, 160 trials employ local administration, and 3 trials do not disclose the ROA information (**Figure 1C**). CNS targeting is the most complicated: intraputaminal (n=18), intracerebral (n=12), intrathecal (n=11), intracisternal magna (ICM, n=9), intrastriatal (n=5), intracerebral (n=4), intraventricular (n=3), 2 intrathalamic (n=2), and intranigral (n=1). Ocular indications are all dosed through local administration, with 37 trials by subretinal, 24 by intravitreal, and 2 by suprachoroidal dosing. For muscle targeting, 22 trials employ systematic dosing while 10 trials apply intramuscular injection. The trial number has significantly increased over the past two decades (**Figure 1D**). The first AAV trial was for treating cystic fibrosis using ​​tgAAV2-CFTR (1), and the first trial using AAV delivery for *in vivo* gene editing was for treating Mucopolysaccharidosis type I by zinc finger nucleases (NCT02702115) (29). There are four approved AAV gene therapy drugs: Glybera (30), Zolgensma (31), Luxturna (32), and Upstaza (33) and one conditional approval Roctavian (34) in the USA and/or EU.

A total of 182 trials disclosed dosing information, and the doses vary largely depending on ROA. For systematic dosing of liver indications, the range is between 5E11 and 1E14 vg/kg. Since vector titers are quantified by each sponsor independently using different primers, probes and assays, it is challenging to perform direct comparison across trials. With this in mind, 14 trials include at least one cohort with a dose higher than 1E14 vg/kg, 6 out of them are targeting muscle indications, and all of them are associated with serious adverse events. Another 6 trials are associated with Zolgensma, which uses self-complementary AAV9 and have been approved as a commercialized product (35). The doses for ocular indications range from 2E8 to 5E11 vg/eye, while 2E10 to 1.1E14 vg for CNS indications. The highest doses for LSD trials are 1.1E14 vg/kg by intravenous injection, 1E14 vg in total by local injection (intraputaminal, intrastriatal, intracerebral), or 1.1E11 vg/g of the brain by ICM injection. A total of 242 trials disclosed serotype information, with 72 of them using AAV2. Other common serotypes are AAV9 (n=36), AAV8 (n=27), AAV5 (n=22), AAV1 (n=17), AAVrh10 (n=13), and AAVrh74 (n=11). There are 20 trials using novel engineered capsids instead of wildtype serotypes (**Table 1**). Based on disclosed information, 20 trials (8.3%) use self-complementary AAV (scAAV), while others use the single-stranded AAV (ssAAV).

**Table 1:** Clinical trials using engineered AAV capsids.

Disease	Trial ID	Drug	Sponsor	Serotype
Achromatopsia	NCT02599922	AGTC-401	AGTC	AAV2tYF
Choroideremia	NCT04483440	4D-110	4DMT	4D-R100
Diabetic macular edema	NCT04418427	ADVM-022	Adverum	AAV2.7m8
LHON	NCT02161380	scAAV2-P1ND4v2	University of Miami	AAV2tYF
Neovascular AMD	NCT05197270	4D-150	4DMT	4D-R100
Neovascular AMD	NCT03748784	ADVM-022	Adverum	AAV2.7m8
RP	NCT03326336	GS030	GenSight Biologics	AAV2.7m8
X-linked retinoschisis	NCT02416622	BIIB-087	AGTC	AAV2tYF
XLRP	NCT04517149	4D-125	4DMT	4D-R100
XLRP	NCT03316560	AGTC-501	AGTC	AAV2tYF
CF	NCT05248230	4D-710	4DMT	4D-A101
Wilson's disease	NCT04537377	VTX-801	Vivet Therapeutics	Anc80
DMD	NCT00428935	d3990	Nationwide Children's Hospital	AAV2.5
Fabry	NCT04519749	4D-310	4DMT	4D-A101
Gaucher	NCT05324943	FLT201	Freeline Therapeutics	AAVS3
MMA	NCT04581785	LB-001	LogicBio	LK03
Hemophilia A	NCT03003533	SPK-8011	Spark Therapeutics	AAV-LK03
Hemophilia B	NCT03369444	FLT180a	University College London	AAVS3
Hemophilia B	NCT02484092	SPK-9001	Spark Therapeutics	AAV-Spark100
Hemophilia B	NCT03307980	SPK-9001	Spark Therapeutics	AAV-Spark100

LHON, Leber hereditary optic neuropathy; XLRP, X-linked retinitis pigmentosa; DMD, Duchenne muscular dystrophy; MMA, methylmalonic acidemia.

### Immunosuppressant usage

**Figure 2:**
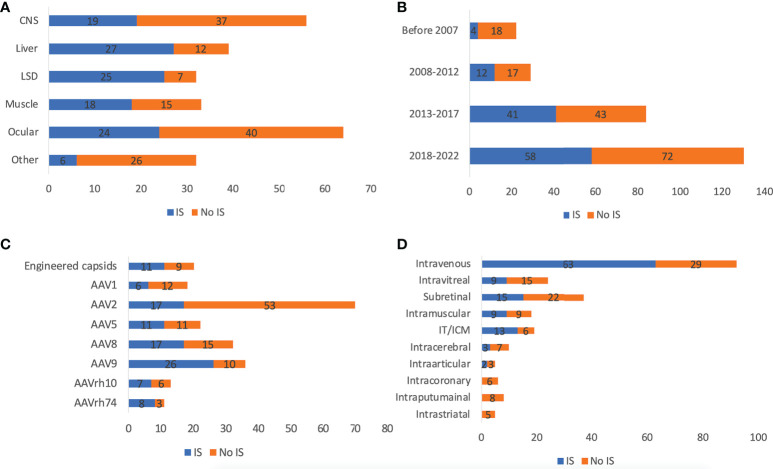
Immunosuppressants usage of 255 AAV clinical trials by disease indications **(A)**, timeframe **(B)**, serotypes **(C)**, and ROAs **(D)**. ROA and serotypes associated with <10 trials are not included in the figure. IT and ICM were combined due to the high similarity.

As shown in **Figure 2**, 46.6% (n=118) of trials applied immunosuppressants (prophylactic or reactive). Most trials used corticosteroids, while Sirolimus and Rituximab^®^ were also frequently used. The use of immunosuppressants also depends on the target tissue rAAV delivered to. Only 33.9% of CNS and 37.5% of ocular-directed trials employed immunosuppressants, possibly due to the immune-privileged status of CNS and retina. In contrast, most muscle, liver, and LSD trials employed immunosuppressants (54.5%-78.1%). In terms of ROA, there was also a high variability. 68.5% of trials using intravenous delivery employed immunosuppressants, similarly, 68.4% of trials using CSF delivery (intrathecal/ICM) used immunosuppressants. Other local CNS injections (intraputaminal, intrastriatal, intracerebral) had at most 30% immunosuppressant usage. As time went by, immunosuppressant usage became more frequent from 18.2% before 2007 to 44.6% between 2018 and 2022. This could be explained by a better understanding of AAV immunology through clinical observations over the years. Among all serotypes, AAVrh74 trials were the most frequent user of immunosuppressants (72.7%, 8/11), followed by AAV9 (72.2%, 26/36), AAV8 (53.1%, 17/32) and AAV5 (50.0%, 11/22). Only 24.3% (17/70) of AAV2 trials employed immunosuppressant, which could be partially explained by the fact that AAV2 was most frequently chosen for ocular diseases.

### Antibody screening

**Figure 3:**
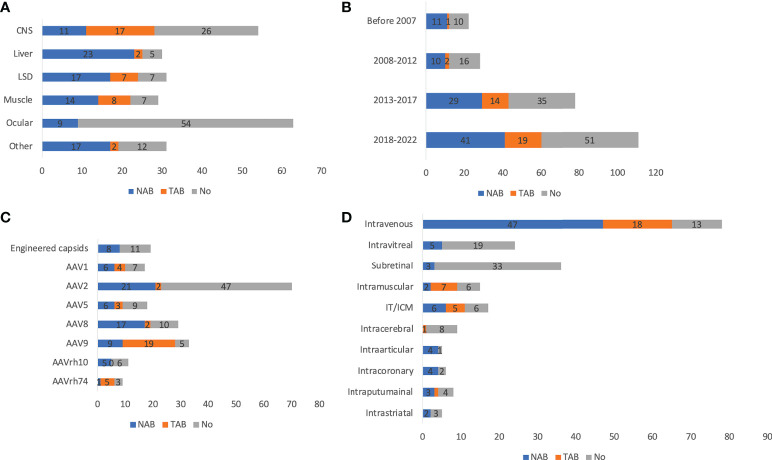
Antibody assays of 255 AAV clinical trials by disease indications **(A)**, timeframe **(B)**, serotypes **(C)**, and ROAs **(D)**. ROA and serotypes associated with <10 trials are not included in the figure. IT and ICM were combined due to the high similarity. Trials that indicated usage of antibody assays but did not specify types of assays are not included.

There was not much fluctuation in usage of antibody screening for patient enrollment over time, from 56.5% before 2007 to 58.5% between 2018 and 2022 (**Figure 3B**). There was an increase in the usage of total antibody assay (TAB) from 4.3% before 2007 to 15.0% between 2018 and 2022. This could be partially explained by the ease of use and higher reliability of TAB than neutralizing antibody assay (NAB). The antibody screening varied greatly by disease indications (**Figure 3A**). CNS and ocular trials were relatively low: 53.6% and 15.6%, respectively. In contrast, liver, LSD, and muscle trials were generally high: 87.5%, 78.8%, and 78.1%, respectively. Another interesting finding is that while NAB was more frequently used in other disease categories (60-100% of those used NAB or TAB), TAB was more frequently used in the CNS trials (59.3%). Among all serotypes, 86.1% of AAV9 trials used antibody screening, followed by AAV8 (67.7%) (**Figure 3C**). In contrast, only 33.8% of AAV2 trials had antibody screening, which could be explained by the frequent usage of AAV2 in ocular diseases. Only 55.0% of trials using engineered capsids had antibody screening. While NAB was more frequently chosen for other serotypes, TAB was the predominant choice for AAV9 (67.9%). In terms of ROA, 86.2% of intravenous delivery employed antibody screening, followed by intraarticular (80%), intracoronary (66.7%), IT/ICM (66.7%), and intramuscular (62.5%) (**Figure 3D**). In contrast, intravitreal (20.8%), subretinal (10.8%), and intracerebral (20.0%) were low. In these disease categories, NAB was the dominant choice except for intramuscular (NAB 22.2% *vs* TAB 77.8%). The titer threshold for excluding patients varied significantly among trials: NAB (1:1 to 1:1,200), TAB (1:5 to 1:1,600). What makes it more difficult to perform cross-trial comparisons is that the methods used for these assays varied significantly (36). In addition, the *in vitro* biological activity of the vectors may also contribute to the wide range of NAB thresholds.

### Toxicity and adverse events

**Figure 4:**
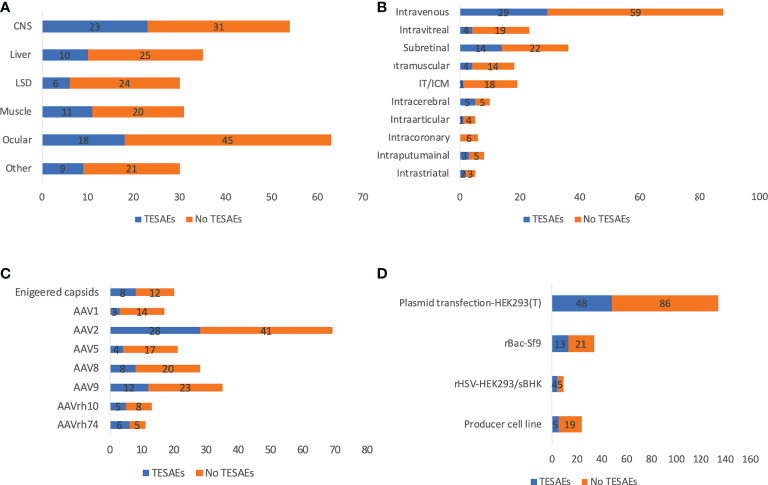
Reported TESAEs of 255 AAV clinical trials by disease indications **(A)**, ROAs **(B)**, serotypes **(C)**, and manufacturing systems **(D)**. ROA and serotypes associated with <10 trials are not included in the figure. IT and ICM were combined due to the high similarity. Trials that initiated recently are not included for analysis.

There were a total of 11 reported patient deaths across 8 trials, with the Audentes trial for X-linked myotubular myopathy having the highest number of patient deaths (n=4), potentially due to hepatotoxicity. The disease indication, ROA, serotype, and production system of these 8 trials are summarized in **Table 2**. It is worthwhile to mention that whether these patient deaths are vector-related is unclear in most cases. Due to the small sample size and lack of detailed reports, it is difficult to make conclusions on patient deaths in AAV clinical trials. In addition, it was recently reported that two patient deaths due to hepatotoxicity after receiving a commercialized gene therapy drug, Zolgensma (44). There were 30 clinical holds, 18 of which were due to adverse events observed in the respective clinical trials. Others were due to CMC issues, toxicity findings in preclinical studies or other relevant clinical studies, and delivery devices. The most prominent TESAEs were hepatotoxicity (45), TMA (46), and neurotoxicity (41), while DRG toxicity attracted much attention recently (39, 47). Among all disease categories, LSD seemed to be the safest, with the least risk of TESAEs (20.0%), while CNS had the highest risk (42.6%) (**Figure 4A**). Among all ROAs, IT/ICM seemed to be the safest (5.3%), while local CNS injections had relatively high risk (37.5-50.0%). Interestingly, intravitreal had a lower risk of TESAEs than subretinal (17.4% *vs* 38.9%), which could be explained by the invasiveness of subretinal injections (**Figure 4B**). In terms of serotypes, AAV1 and AAV5 seemed to be safer, with a risk of TESAEs at 17.6% and 19.0%, respectively (**Figure 4C**).

**Table 2:** Patient deaths that occurred in AAV-related clinical trials.

Disease	Trial ID	Drug name	Serotype	ROA	Production	Patient deaths	References
DMD	NCT03362502	PF-06939926	AAV9	Intravenous	Undisclosed	1	37
ALS	N/A	AAV-miR-SOD1	AAVrh10	Intrathecal	HEK293	1	38
MPS IIIA	NCT03612869	SAF-302	AAVrh10	Intracerebral	HEK293	1	39
GAN	NCT02362438	TSHA-120	AAV9	Intrathecal	HEK293	1	40
GM2 gangliosidosis	NCT04798235	TSHA-101	AAV9	Intrathecal	HEK293	1	41
SMA	NCT03461289	Zolgensma	AAV9	Intravenous	HEK293	1*	42
XLMTM	NCT03199469	AT132	AAV8	Intravenous	HEK293	4	43, 44

Two patient deaths were recently reported when Zolgensma is commercially used, and no detailed information was released yet (45). One patient death in a clinical trial for arthritis was excluded because it was deemed not be related with AAV (46). ALS, amyotrophic lateral sclerosis; GAN, giant axonal neuropathy; SMA, spinal muscular atrophy.

### Durability

**Figure 5:**
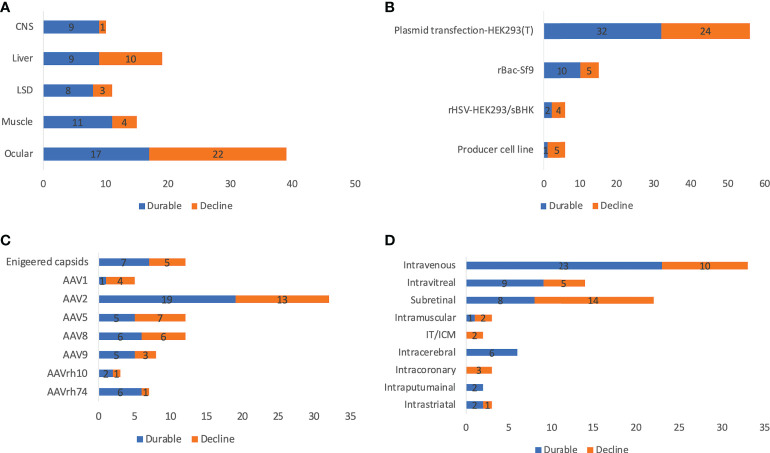
Reported durability of 255 AAV clinical trials by disease indications **(A)**, manufacturing systems **(B)**, serotypes **(C)**, and ROAs **(D)**. ROA and serotypes associated with <10 trials are not included in the figure. IT and ICM were combined due to the high similarity. Trials that initiated recently or did not report efficacy and transgene expression data are not included for analysis.

Long-term clinical data from AAV trials showed incidences of declining transgene expression over time, which may be due to immune response and vector dilution. Declines in transgene expression occur either rapidly or gradually, indicating two distinct mechanisms (presumably acute immune response *vs* vector dilution). In this study, ‘durable’ was defined as sustained transgene expression or clinical efficacy in all patients during the latest follow-up visit, otherwise it was defined as ‘decline’. Notably, sometimes it is difficult to distinguish lack of initial efficacy and declining transgene expression because some trials observed no efficacy in the first follow-up. Up to 90.0% of CNS and 73.3% of muscle diseases (only those that had publicly available durability data counted) achieved durable transgene expression, in spite of the limited follow-up time and relatively small sample size (**Figure 5A**). In contrast, only 47.4% of liver trials achieved durability, which can be explained by higher risk of immunogenicity due to systemic administration and active cell divisions of hepatocytes. Only 43.6% of ocular trials achieved durable efficacy, which is counterintuitive because the retina is usually seen as an immune-privileged compartment (48). In general, there was significant inter-patient variability, possibly due to differences in age, genetic background, disease progression, or immune system. This highlights the need of careful patient selection in designing clinical trials. Further, there was no clear correlation between dose and durability even within individual trials. For example, in one trial (RGX-314 for wet AMD), lower doses achieved better durability. This may be because although high dose leads to higher initial transgene expression for decline to begin with, it also causes higher risk of immune response. It is expected to be necessary to identify an optimal balance between high dose and low immunogenicity. Trials using engineered capsids (LK03 for hemophilia A, AAV-Spark100 and AAVS3 for hemophilia B) seemed to achieve better durability even with relatively low doses. However, in general, there is no clear pattern in terms of durability between different serotypes (**Figure 5C**). One may expect subretinal injections would achieve better durability because AAV is injected into an immune-privileged compartment (49). However, there was no evidence supporting it (intravitreal 64.3% *vs* subretinal 36.4%) (**Figure 5D**).

### Production system

**Figure 6:**
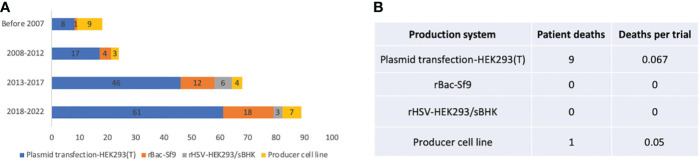
Manufacturing systems of 255 AAV clinical trials. **(A)** System usage in different time periods. **(B)** Patient deaths in trials using different manufacturing systems. Trials that did not report manufacturing systems are not included for analysis.

Plasmid transfection in HEK293 or HEK293T cells became the predominant AAV production system since 2007, while the producer cell line was more frequently used before 2007. There is an increase in the rBac-Sf9 system from 5.6% before 2007 to 20.2% between 2018 and 2022 (**Figure 6A**). The pros and cons of each system and system used by different companies or institutes are summarized in **Table 3**. The rBac/Sf9 system has advantages in yield, empty/full ratio, and scalability over other systems (50, 51). Yet, one study showed that AAV produced by the rBac/Sf9 system had a higher degree of truncated and unresolved species than those generated by the HEK293 system (52). Another study showed that the rBac/Sf9 system may lead to difference in post-translational modifications, host cell protein impurity profile, and methylation status of capsid proteins, as well as reduced potency compared to the HEK293 system (53). However, the differences observed in this study was not substantial, and the vectors generated from the two systems were not of similar quality. Moreover, it was reported that by modulating ratio of VP1/2/3 capsid composition in the rBac/Sf9 system, the potency of AAV vectors could be significantly improved to be comparable to the HEK293 system (54). All of these may have implications for AAV immunogenicity and toxicity in clinical studies. Nonetheless, no difference in risk of TESAEs were found between trials using rBac/Sf9 (38.2%) or HEK293 (35.8%) (**Figure 4D**). Also, a total of 9 patient deaths occurred in 134 AAV trials using the HEK293 system, while there is one individual death for the producer cell line system (n=24) (**Figure 6B**). The impact of the production system on durability of transgene expression was also assessed. HEK293 and rBac-Sf9 systems had comparable results: 58.2% and 66.7% of trials achieved durable efficacy, respectively (**Figure 5B**). Interestingly, producer cell line and rHSV systems had only 16.7% and 33.3% of trials achieving durability. Admittedly, the sample size is small.

**Table 3:** Comparison of AAV manufacturing systems.

Manufacturing system	Pros & Cons	Companies or institutes
Plasmid transfection in HEK293/HEK293T	Yield (++), Full/Empty ratio (+), Scalability (+), Timeline (+++)	HEK293: Abbvie, Abeona Therapeutics, Amicus Therapeutics, ASC Therapeutics, AskBio, Audentes, Avigen, Capsida, Ceregene, Children's Hospital of Philadelphia, Editas, Forge Biologics, GenSight Biologics, Harvard Gene Therapy Initiative, Homology Medicines, Lonza*, Lysogene, MeiraGTx*, Nationwide Children's Hospital, Neurogene*, NeuroLogix, Novartis Gene Therapies, Passage Bio, REGENX Bio, Rocket Pharma, Spark Therapeutics, Sarepta, Sio Gene Therapies, Taysha Gene Therapies, University of Florida, University of Pennsylvania, 4DMT
		HEK293T: Freeline Therapeutics, MeiraGTx*, Genethon, Nantes University Hospital, Nightstar Therapeutics, St. Jude Children's Research Hospital, University College London, Weill Medical College of Cornell University
Baculovirus in Sf9	Yield (+++), Full/Empty ratio (+++), Scalability (+++), Timeline (++)	Adverum, Amsterdam Molecular Therapeutics, Avirmax, BioMarin, Frontera Therapeutics, Neurogene*, Pfizer, Prevail Therapeutics, Lonza*, Sangamo Therapeutics, UniQure, Voyager Therapeutics
HSV in HEK293 or sBHK	Yield (++), Full/Empty ratio (++), Scalability (++), Timeline (++)	AGTC*, Neurophth, Solid Biosciences
Producer cell line	Yield (++), Full/Empty ratio (+), Scalability (+++), Timeline (+)	Atsena Therapeutics, Celladon Corporation, Targeted Genetics Corporation, Ultragenyx

HSV, Herpes simplex virus; sBHK, suspension baby hamster kidney cells. * indicates companies or institutes reporting multiple systems.

+ indicates the level of strength.

## Discussions

Immune responses against AAV pose significant challenges for gene therapy development, impacting patient enrollment, toxicity profile, and durability of efficacy. Systematic analysis of currently available clinical data is an important initial step to overcome immune responses against AAV, which could be empowered by accumulating data and more open data-sharing. This study analyzed multiple aspects of 255 AAV clinical trials, providing a knowledge basis for future preclinical and clinical study design. Previous studies have shown that pre-existing antibodies could significantly inhibit AAV transduction (55) and activate memory cells (56). Therefore, pre-existing antibodies exclude a large portion of patients from receiving potentially life-saving treatments due to the relatively high prevalence among human population. It becomes a bigger issue when targeting diseases like Parkinson’s disease because as people age, they would have a higher likelihood to be exposed to AAV. One approach is to use wildtype or engineered capsids that have less seropositive prevalence in human population (53, 54). Another option is using local administration, which is expected to have a reduced risk of interactions between AAV and antibodies (55). Other methods include plasmapheresis (57, 58), saline flushing (59), IgG proteases (60), capsid engineering (61–63), and lipid nanoparticle encapsulated rapamycin (64), most of which are under preclinical development. In clinical settings, a more frequently applied method is antibody screening during patient enrollment. NAB directly measures antibodies that neutralize AAV vectors and inhibit transduction. However, as a cell-based assay, NAB has a higher variability due to differences in cell line maintenance, report gene choices, and multiplicity of infections used (36). Other drawbacks of NAB include the omission of the contribution of non-neutralizing antibodies to immune responses and logistic difficulty when compared with TAB. Possibly due to the aforementioned reasons, this study observed an increased percentage of TAB usage over time. Another key aspect of immune response against AAV in clinical setting is toxicity, evidenced by multiple incidences of hepatotoxicity (44–46, 65), TMA (25, 26), MRI abnormality (66), and DRG toxicity (39, 47). Considering the complexity in chemistry manufacturing and controls (CMC) of recombinant virus products, it is not feasible to directly compare across products from different sponsors. However, a significant number of trials showed a positive correlation between dose and efficacy, meanwhile high doses may lead to a higher risk of complement activation (25, 26), liver enzyme elevations (67), and other adverse effects (66). In many cases, liver enzyme elevations and complement activation can be alleviated by immunosuppressants (67), but not always (46). DRG toxicity has also been reported to be dose-dependent in multiple large animal studies (68). Fortunately, this was less frequently observed in clinical studies, expect two trials for amyotrophic lateral sclerosis (39) and giant axonal neuropathy (41). One potential reason could be species differences. Notably, researchers have developed a potential strategy to reduce the DRG toxicity by silencing the transgene expression in DRG *via* microRNA (69).

One of the advantages for AAV gene therapy is seen as relatively long-lasting transgene expression, which is achieved mainly through episomal AAV (70, 71). Previous studies showed sustained efficacy for 3 years or longer periods (72, 73). However, many clinical studies observed reduced transgene expression over time, for instance, in some trials for Hemophilia A. In addition to vector dilution, the durability of efficacy can also be impacted by CTL-mediated elimination of transgene-expressing cells (74) and promoter silencing (75). This situation is further exacerbated by the fact that repeated dosing is challenging due to immune responses against AAV (8). In this study, there is high variability in durability across trials, depending on multiple factors, including disease indications, serotypes, ROAs, doses, AAV genome sequences, and genetic background of individual patients. It has been shown that muscle gene transfer could achieve sustained efficacy through inducing immune tolerance by regulatory T cells (76–78). In this study, 73.3% muscular trials achieved durable transgene expression (**Figure 5A**). However, although there is also clinical evidence of induction of immune tolerance through liver gene transfer (79), only 47.4% of liver trials achieved durable efficacy. This may be partially explained by the more rapid hepatocyte turnover and vector dilution, as well as more antigen presentation due to systemic administration. CNS and retina are usually seen as ‘immune-privileged’ compartments, and gene transfer to CNS and retina are expected to achieve more durable efficacy. Although 90% of CNS achieved durable transgene expression, only 43.6% of ocular trials observed sustained efficacy. This highlighted the complexity in designing ocular trials and the need to carefully select dose, ROA, and patients (genetic background, disease progression, age). Overall, the durability of AAV gene therapies is reasonably good, although careful patient selection and optimized design are warranted.

In addition to immune responses, AAV toxicity can result from potential insertional mutagenesis. rAAV exists mainly as an episome, but still be able to integrate into host chromosome DNA (80). It was estimated that intravenous administration may deliver >100 vector copies per hepatocyte on average (81). With the robust metabolism and strong regeneration activity of hepatocytes, there are concerns about vector genome insertion and oncogenesis. Tumorigenesis events are documented in rodent studies but shown to be less frequent in large animals (71, 82–86), indicating a species dependence. The integration risk also seems to be serotype-dependent. One mouse study using AAV9 observed hepatocellular carcinoma (87), while a similar study using AAV5 did not (88). In addition, the risk also depends on promoter choice as shown in a murine study (86). Currently, only one patient developed a liver tumor (NCT03569891) and finally showed not to be related with AAV dosing (89). Improving the vector potency and specificity by engineering may significantly decrease the required dose, which reduces the risk of genome insertion. Also, exploring the genome design to accelerate the circularization kinetics may help reduce the exposure of the linear genome to double strand breaks, which may reduce the insertion events. In light of this, the FDA recently organized a Cellular, Tissue, and Gene Therapies Advisory Committee (CTGAC) meeting to address AAV toxicity issues, including AAV integration risk. In summary, the insertional mutagenesis risk of AAV is relatively low in the context of currently available clinical data, but it should be monitored in the long-term follow up studies as recommended by the FDA.

Compared with small or large molecule drugs, another crucial challenge for gene therapy is translatability. Remarkable efficacy has been observed in many small animal studies, while the translatability to large animal and clinical trials are limited. For instance, an engineered capsid selected from a C57BL/6J mice strain penetrates the blood brain barrier efficiently (90). However, the receptor for this selected capsid, PHP.B, only exists in some mouse strains but not others or NHPs (91). There is a high demand for designing and screening better capsids to enter target cells or tissue more efficiently and specifically. However, due to the complexity and differences of blood circulation, cell and tissue types, cell surface protein patterns related with attachment and binding, many novel capsids work well only in certain animal models (91, 92). Understanding the species differences and developing new models for mimicking the specific questions to solve will enhance the translatability in the future. Another example is the vector penetration for ocular indications by intravitreal injection. Human eye structure has the inner limiting membrane, which brings extra physical barriers than mouse species. In addition, the cross-species translatability issue also pose significant challenges for predicting immune responses in clinical studies from preclinical data. This holds especially true for T cell responses as expansion of capsid-specific CD8^+^ T cells was observed in humans (93), but not mice (94), dogs (95), and NHPs (96). Thus, it is critical to develop suitable animal models to recapitulate immune responses against AAV in humans.

## Data availability statement

The original contributions presented in the study are included in the article/**Supplementary Material**. Further inquiries can be directed to the corresponding author.

## Author contributions

WS and LO designed the study, collected data, performed data analysis, and wrote and revised the manuscript. SL revised the manuscript. All authors contributed to the article and approved the submitted version.

## Acknowledgments

The authors would like to thank Drs. Jingmin Zhou and Patty Biezonski from Genemagic Biosciences for reviewing the manuscript.

## Conflict of interest

LO is an employee of Genemagic Biosciences, WS is an employee of Obio Technologies, and SL is an employee of Avirmax.

## Publisher’s note

All claims expressed in this article are solely those of the authors and do not necessarily represent those of their affiliated organizations, or those of the publisher, the editors and the reviewers. Any product that may be evaluated in this article, or claim that may be made by its manufacturer, is not guaranteed or endorsed by the publisher.
