# Corrigendum: rAAV immunogenicity, toxicity, and durability in 255 clinical trials: A meta-analysis

**DOI:** 10.3389/fimmu.2022.1104646

**Published:** 2023-01-18

**Authors:** Weiran Shen, Shengjiang Liu, Li Ou

**Affiliations:** ^1^ Obio Technologies, Shanghai, China; ^2^ Avirmax Inc, Hayward, CA, United States; ^3^ Genemagic Biosciences, Wallingford, PA, United States; ^4^ Department of Pediatrics, University of Minnesota, Minneapolis, MN, United States

**Keywords:** rAAV, clinical trials, immunogenicity, toxicity, neutralizing antibodies, immunosuppressants, capsids, gene therapy

In the published article, there was an error in **Table 2** as published. Row 1, column 6 of the table should be “undisclosed” instead of “rBac-Sf9”. Furthermore, the row “Inflammatory arthritis” was removed as the death that occurred in that clinical trial was not AAV-related. The corrected **Table 2** and its caption “Table 2. Patient deaths that occurred in AAV-related clinical trials” appear below.

**Table 2:** Patient deaths that occurred in AAV-related clinical trials.

Disease	Trial ID	Drug name	Serotype	ROA	Production	Patient deaths	References
DMD	NCT03362502	PF-06939926	AAV9	Intravenous	Undisclosed	1	37
ALS	N/A	AAV-miR-SOD1	AAVrh10	Intrathecal	HEK293	1	38
MPS IIIA	NCT03612869	SAF-302	AAVrh10	Intracerebral	HEK293	1	39
GAN	NCT02362438	TSHA-120	AAV9	Intrathecal	HEK293	1	40
GM2 gangliosidosis	NCT04798235	TSHA-101	AAV9	Intrathecal	HEK293	1	41
SMA	NCT03461289	Zolgensma	AAV9	Intravenous	HEK293	1*	42
XLMTM	NCT03199469	AT132	AAV8	Intravenous	HEK293	4	43, 44

Two patient deaths were recently reported when Zolgensma is commercially used, and no detailed information was released yet (45). One patient death in a clinical trial for arthritis was excluded because it was deemed not be related with AAV (46). ALS, amyotrophic lateral sclerosis; GAN, giant axonal neuropathy; SMA, spinal muscular atrophy.

In the published article, there was an error in the legend for **Table 2** as published. We have removed one row in **Table 2**, so the Table legend needs to be updated accordingly. The corrected legend appears below.

“Two patient deaths were recently reported when Zolgensma is commercially used, and no detailed information was released yet (45). One patient death in a clinical trial for arthritis was excluded because it was deemed not be related with AAV (46). ALS, amyotrophic lateral sclerosis; GAN, giant axonal neuropathy; SMA, spinal muscular atrophy.”

In the published article, there was an error in **Figure 6** as published. In **Figure 6B**, both column “patient deaths” and “deaths per trial” for rBac-Sf9 should be 0. The corrected **Figure 6** and its caption “Figure 6. Manufacturing systems of 255 AAV clinical trials” appear below.

**Figure 6:**
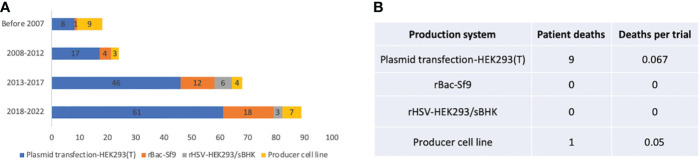
Manufacturing systems of 255 AAV clinical trials. **(A)** System usage in different time periods. **(B)** Patient deaths in trials using different manufacturing systems. Trials that did not report manufacturing systems are not included for analysis.

In the published article, there was an error. There was no patient death for the rBac/Sf9 system. Our information source on one trial was wrong.

A correction has been made to **Results**, *Production system*. This sentence previously stated:

“Also, a total of 9 patient deaths occurred in 134 AAV trials using the HEK293 system, while there is one individual death for both rBac/Sf9 system (n=36) and producer cell line system (n=24) (**Figure 6B**).”

The corrected sentence appears below:

“Also, a total of 9 patient deaths occurred in 134 AAV trials using the HEK293 system, while there is one individual death for the producer cell line system (n=24) (**Figure 6B**).”

The authors apologize for these errors and state that they do not change the scientific conclusions of the article in any way. The original article has been updated.

