# Supplementary figures and images for: rAAV immunogenicity, toxicity, and durability in 255 clinical trials: A meta-analysis
Source: Front Immunol. 2022 Oct 27;13:1001263. doi: 10.3389/fimmu.2022.1001263 (PMC9647052; doi:10.3389/fimmu.2022.1001263)

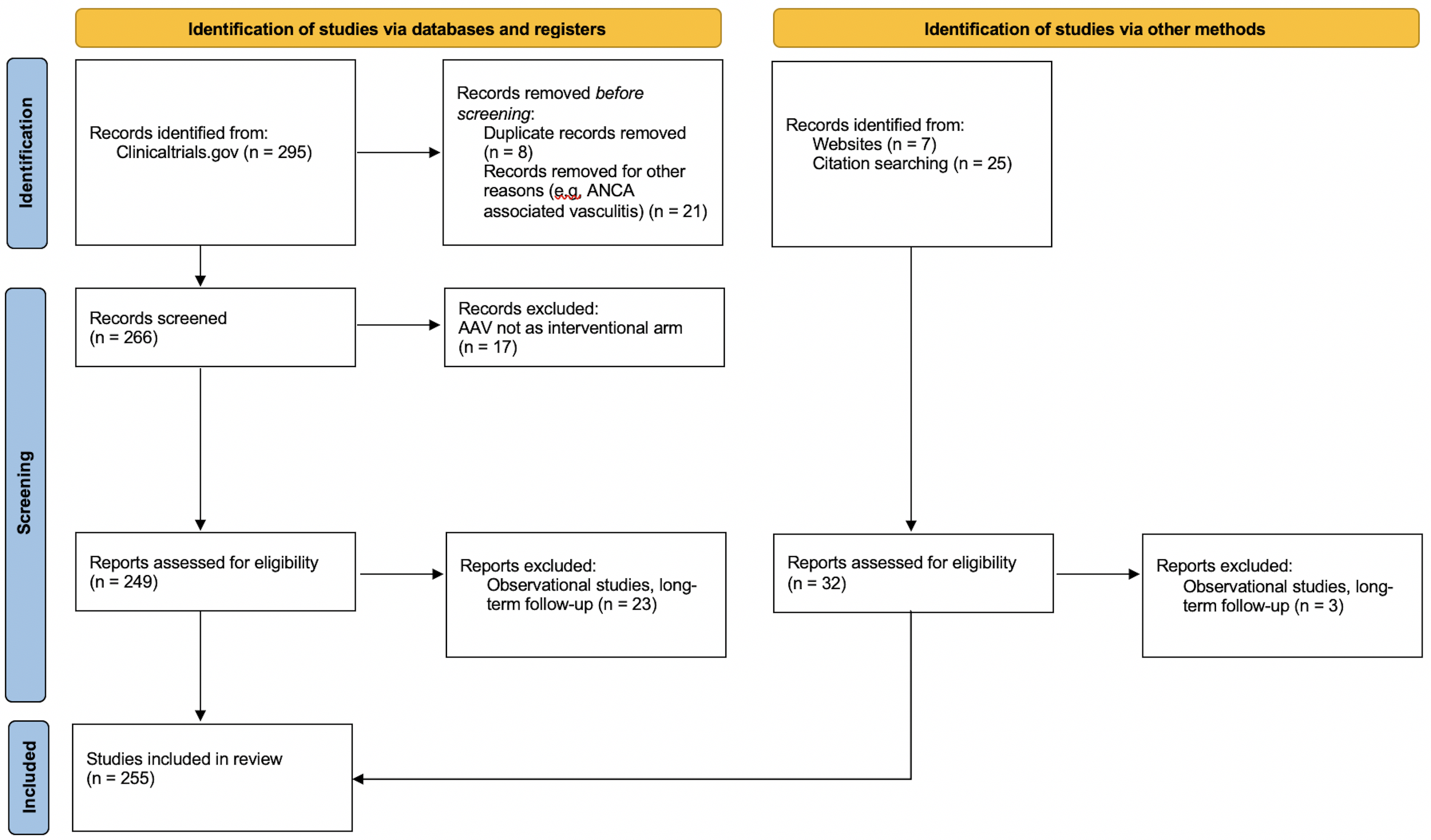

Supplement: Supplementary file 2 [file Image_1.jpeg]
